# Developing a partnership to improve health care delivery to children <18 years with cancer and blood disorders in the English-speaking Caribbean: lessons from the SickKids-Caribbean Initiative (SCI)

**DOI:** 10.1016/j.lana.2023.100592

**Published:** 2023-09-11

**Authors:** Michelle Reece-Mills, Curt Bodkyn, Jo-Anna B. Baxter, Upton Allen, Cheryl Alexis, Chantelle Browne-Farmer, Jenna Craig, Stephanie de Young, Avram Denburg, Kevon Dindial, Bonnie Fleming-Carroll, Tracey Gibson, Sumit Gupta, Jennifer Knight-Madden, Margaret Manley-Kucey, Sharon Mclean-Salmon, Oscar Noel Ocho, Kadine Orrigio, Stanley Read, Corrine Sin Quee, Brian Smith, Minerva Thame, Gilian Wharfe, James A. Whitlock, Stanley Zlotkin, Victor Blanchette

**Affiliations:** a Faculty of Medical Sciences, Department of Child and Adolescent Health, The University of the West Indies, Kingston, Jamaica; b Department of Clinical Medical Sciences, The University of the West Indies, St. Augustine, Trinidad and Tobago; c Centre for Global Child Health, Hospital for Sick Children, Toronto, Canada; d Division of Infectious Diseases, Hospital for Sick Children, Toronto, Canada; e Division of Haematology/Oncology, Hospital for Sick Children, Toronto, Canada; f Institute of Health Policy Management and Evaluation, University of Toronto, Toronto, Canada; g Haematology/Oncology Department, Queen Elizabeth Hospital, Bridgetown, Barbados; h Department of Paediatrics, Queen Elizabeth Hospital, Bridgetown, Barbados; i Department of Paediatrics, Eric Williams Medical Sciences Complex, San Juan, Trinidad and Tobago; j Learning Institute, Hospital for Sick Children, Toronto, Canada; k Lawrence S. Bloomberg Faculty of Nursing, University of Toronto, Toronto, Canada; l Department of Pathology, The University of the West Indies, Mona, Jamaica; m Department of Paediatrics, University of Toronto, Toronto, Canada; n Sickle Cell Unit, Caribbean Institute for Health Research, The University of the West Indies, Kingston, Jamaica; o Hematology/Oncology Unit, Bustamante Hospital for Children, Kingston, Jamaica; p Faculty of Medical Sciences, School of Nursing, The University of the West Indies, St. Augustine, Trinidad and Tobago; q PAHO/WHO Collaborating Centre for Nursing and Midwifery Development in the Caribbean, School of Nursing, The University of the West Indies, St. Augustine, Trinidad and Tobago; r School of Clinical Medicine and Research, The University of the West Indies, Nassau, The Bahamas; s Department of Haematology/Oncology, The University of the West Indies, Kingston, Jamaica

**Keywords:** Caribbean, Oncology, Haematology, Sickle cell disease, Paediatrics, Education, Training, Database, Advocacy

## Abstract

In 2013, the SickKids-Caribbean Initiative (SCI) was formalised among The Hospital for Sick Children in Toronto, Canada, the University of the West Indies, and Ministries of Health in six Caribbean countries (Barbados, The Bahamas, Jamaica, St. Lucia, St. Vincent and the Grenadines, and Trinidad and Tobago). The aim was to improve the outcomes and quality of life of children (<18 years) with cancer and blood disorders in the partner countries. Core activities included filling a human resource gap by training paediatric haematologists/oncologists and specialised registered nurses; improving capacity to diagnose and treat diverse haematology/oncology cases; developing and maintaining paediatric oncology databases; creating ongoing advocacy activities with international agencies, decision makers, and civil society; and establishing an integrated administration, management, and funding structure. We describe core program components, successes, and challenges to inform others seeking to improve health service delivery in a multidisciplinary and complex partnership.

## Background

Outcomes for children with cancer and blood disorders vary globally depending on a country’s health system. In high-income countries (HICs), improvements in prognosis over the past decades have been notable, but have not been observed comparably worldwide.^1^^,^^2^ This is critical, particularly when considering an estimated 80% of paediatric cancer cases and high prevalence of sickle cell disease (SCD) occurring in low- and middle-income countries.^3^, ^4^, ^5^ Constraints to equitable health care include inadequate funding; shortages in appropriately trained healthcare professionals to identify and treat such diseases; limited and/or unreliable access to specialised testing and essential medicines; and lack of registries and case tracking to facilitate appropriate resource planning and policy generation.^2^^,^^6^, ^7^, ^8^

Latin America and the Caribbean have acknowledged health systems inequities, and cancer presents a growing problem.^7^^,^^9^ Comprised of small island countries and territories, the Caribbean predominantly consists of upper-middle-income countries and HICs, yet the general population faces significant barriers to accessing health services.^10^ With respect to cancer and blood disorders, healthcare provision within the public health system is complex and severely affected by inadequate access to medical specialists, infrastructure, and resources.^10^^,^^11^ Among children with cancer, outcomes are worse than those in most HICs.^12^

To address the observed inequalities in childhood cancer and blood disorders worldwide, twinning partnerships, specialised education, and telemedicine are promising, especially when situated within health system-wide efforts to improve care. These can offer healthcare professionals in resource-constrained settings improved access to focussed and continued training, and support in developing treatment and care plans, via the guidance and support of experts in resource-abundant settings.^13^, ^14^, ^15^, ^16^ The development of locally-managed cancer registries that track patient outcomes also helps to understand the burden, guide treatment, and implement appropriate policy.^12^ Dedicated human resource support and advocacy efforts directed at Ministries of Health, stakeholders, and professional and civil societies further support these strategies.

In 2013, healthcare professionals at a tertiary-care paediatric hospital in Toronto, Canada formally partnered with seven Caribbean institutions across six countries (Barbados, The Bahamas, Jamaica, St. Lucia, St. Vincent and the Grenadines, and Trinidad and Tobago), with the primary goal of improving the outcomes and quality of life of children <18 years with cancer and blood disorders. This report outlines the core program components and identifies major successes and challenges, to inform others aiming to develop a multidisciplinary, complex partnership to improve health service delivery and patient outcomes.

## Program timeline, partners, and evolution of the SickKids-Caribbean Initiative (SCI)

SCI emerged in recognition of the need for improved paediatric haematology/oncology expertise and diagnostic service capacity in the partner six Caribbean countries. Inspired by *ad hoc* physician-to-physician consultations on complex cases involving Caribbean children <18 years, the collaboration was facilitated by formal and informal relationships between physicians at the Hospital for Sick Children (SickKids) and the English-speaking Caribbean countries.^17^ Recognising and supporting the potential for improving the health and well-being of children with cancer and blood disorders led to a formal needs assessment and stakeholder engagement activity in 2010. Importantly, this allowed for the identification of the core partners, buy-in from institutional and governmental leadership, and engagement of donors.^17^

**Fig. 1:**
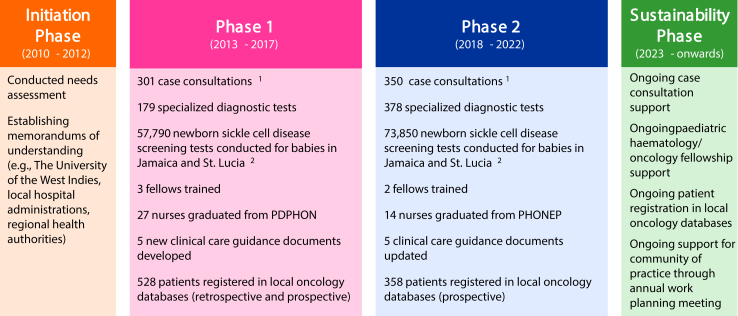
**Phases and key activities within SCI, from inception to current day.**^1^651 case consultations conducted in total to March 31, 2022, including solid tumour (215), leukaemia/lymphoma (208), haematology (119), neuro-oncology (100), and other (9). ^2^Provided by the support of local stakeholders. PDPHON, post-registration diploma in paediatric haematology/oncology nursing.

In 2013, SCI was formally launched as a partnership among the Centre for Global Child Health at SickKids, the University of the West Indies (The UWI), Ministries of Health, and hospitals at seven sites in the six Caribbean countries. From the beginning, clear objectives and governance have grounded SCI, as further described by Manley-Kucey et al.^17^ Briefly, there were two core phases within SCI: phase 1 (2013–2018) focussed on six priorities identified within a needs assessment to develop capacity and phase 2 (2019–2022) built upon phase 1, with a focus on sustainability (Fig. 1). Monitoring and evaluation were integrated across all SCI areas of focus, including a logic model and performance management framework, and a dashboard was maintained and shared with stakeholders on a quarterly basis.^17^ External qualitative reviews were also conducted at the mid- and endpoint of the first two phases of SCI.^18^^,^^19^ Through the generosity of many organisations and individuals in or with ties to the Caribbean, SickKids Foundation provided funding for SCI with 8 million CAD raised for phase 1 and 5 million CAD raised for phase 2. These funds were supplemented by in-kind donations by all partners.

Following phase 2, SCI has transitioned into a phase of long-terms sustainability underpinned by the establishment of an endowment by SickKids Foundation. Caribbean-based leadership has been further engaged, with SickKids continuing to provide administrative structure, access to case consultations and training fellowships, and some support for the Caribbean-administered paediatric oncology databases.

## Core activities

**Fig. 2:**
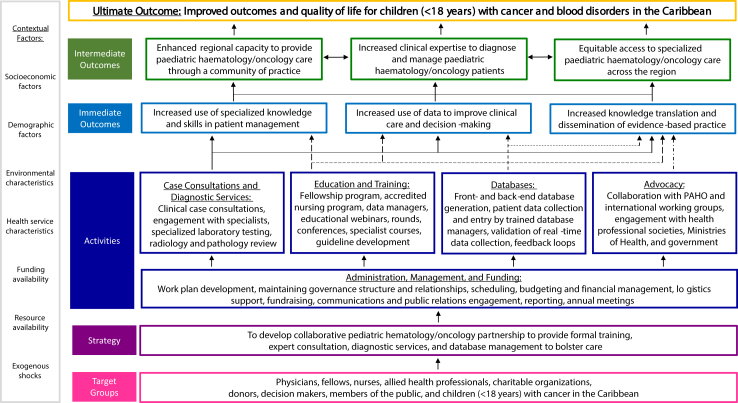
**Framework of activities and outcomes withinSCI.**

**Fig. 3:**
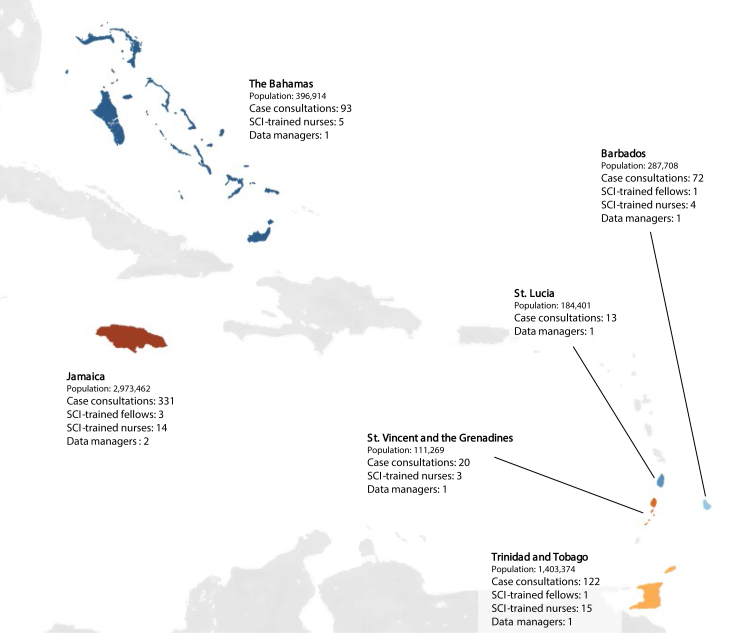
**Distribution of case consultations, SCI-trained paediatric haematology/oncology physicians and nurses, and oncology database managers across the SCI partner countries between2013 and 2022.**

Several activities within SCI (Fig. 2) aimed to improve the outcomes and quality of life for children with cancer and blood disorders in the partner countries. Notably, prior to SCI, there were three specialist paediatric haematologist/oncologists in the partner Caribbean sites (one each in The Bahamas, Barbados, and Trinidad and Tobago). This had not changed in the decade prior, and there were no specialist paediatric haematology/oncology nurses. Described in further detail elsewhere, the core activities included (Fig. 3):

• Physician-to-physician case consultations for complex paediatric haematology/oncology cases^20^;
• Specialised education and training of physicians and nurses in paediatric haematology/oncology and SCD, including newborn screening^21^;
• Development of local paediatric oncology databases, including retrospective and prospective data collection, to reliably monitor incidence, treatments, and outcomes^12^^,^^22^; and
• Advocacy efforts and stakeholder engagement to raise awareness of the burden of paediatric haematology/oncology in the Caribbean, and the opportunity for outcome improvement.^23^

Underpinning all activities was a multi-level governance model, including administration, management, and funding support to ensure operational flow.^17^ Research projects were also integrated within SCI, such as evaluating access to childhood cancer medicines.^8^ In external evaluations, these collective efforts have been described as highly relevant to the needs of partner countries and individuals engaged in SCI, thus filling a gap in the diagnosis, treatment, and care of children with cancer and blood disorders in the partner countries.^18^^,^^19^

## Themes identified within SCI

Within the SCI partnership, four cross-cutting themes emerged: the formation of communities, in lieu of practicing in isolation; building local capacity, as opposed to ‘parachuting in’ with pre-identified priorities; a transition from supporting (i.e., provision of resources/infrastructure) to strengthening (i.e., changing organizational structures, relationships, and behaviour, and promoting more effective use of resources to improve the delivery of health services) the health system; and efforts to optimise practices in a setting-appropriate manner, rather than maintaining the status quo. These themes were identified internally by reviewing past internal and external SCI reports, and were approved by the SCI Research, Policy, and Advocacy Working Group. The following discussion of these themes highlights some of the key successes and challenges.

### Communities versus silos

The formation of formal and informal communities was an important consequence of SCI. As noted, prior to the partnership, three paediatric haematologists/oncologists practiced in the partner countries. Through SCI fellowships, this has increased to seven; 41 nurses have also received specialist paediatric haematology/oncology training.^21^ In this way, SCI has helped to formalise the development of a community of practice, and fostered Caribbean-Caribbean, SickKids-Caribbean, and SCI-international connections. Among the Caribbean specialists, both formal and informal collaborations have developed, given the shared experiences of providing patient care in a resource-limited setting to improve patient outcomes, and with the formal creation of a paediatric sub-committee of the Caribbean Association for Oncology and Haematology (CAOH). The latter is an important step, as CAOH is recognised by the Caribbean Community and Common Market (CARICOM) for improving oncology services within the Caribbean region. The review of complex cases and diagnostic support offered through the case consultations was an important contributor to SickKids-Caribbean connections, as SickKids haematological/oncological experts could be consulted and provide critical guidance on management plans. With respect to generation of international connections, there are several examples to draw upon, from capacity building and efforts to raise the profile of childhood cancer and blood disorders in the Caribbean, as demonstrated through partnerships with the Pan-American Health Organization (PAHO) as part of the Childhood Cancer Working Group for Latin America and the Caribbean,^24^ to working with the American Society of Haematology’s Children’s International Consortium on Acute Leukaemia (C-ICAL) to improve outcomes in children with acute lymphoblastic leukaemia. Building capacity within countries also led to the development of multidisciplinary teams, including doctors, nurses, and allied health professionals, which fostered a sense of ownership and shared responsibility in driving the progress of care for these children.

### Building local capacity versus parachuting in

Building local ownership and initiative was a priority within SCI, and the sustainability of the project has been a core component of its mandate. To support the agency and development of the Caribbean-based healthcare professionals, establishing trust and open communication early on was critical. While this took time, including individuals from both the Caribbean and Canada in the governance structure and decision-making was felt to be critical to success, as were yearly in-person group priority setting exercises. Routine communications (e.g., e-newsletter, reports) also helped to maintain engagement and ensure that partners and stakeholders were aware of the status of the diverse activities underway. Given that the resources in the partner countries differed substantially from those accessible in HICs, ensuring that diagnostic and treatment plans aligned with setting-based realities were very important. Of the different core activities, the locally-built and initiative-informing databases, development of clinical and supportive care guidance documents, and strengthening of local educators and preceptors are examples of how local capacity was supported and facilitated.

### Strengthening versus supporting health systems

Within health systems, resources and infrastructure must first be in place before increased attention is focussed on strengthening efforts.^25^ Following the needs assessment, it was clear early on that certain resources and infrastructure requirements were needed to adequately support program objectives, thus providing or facilitating access to such inputs became an important part of phase 1. One example of the transition from supporting to strengthening was setting up telemedicine facilities at SCI sites.^26^ Whether for case consultations, rounds, trainings, or meetings, these facilities improved connectivity and enabled equitable and efficient health service delivery. In both phase 1 and 2, there was emphasis on strengthening the healthcare workforce by facilitating continued professional development and increasing human resources. The gains in phase 1 meant that phase 2 could concentrate more on improving high-quality service delivery and effective use of resources over the long-term. Another example of the transition from supporting to strengthening was the locally developed paediatric oncology databases, created to support the collection of health information and outcomes and implement findings to strengthen practices. Due to diverse barriers, not all resources executed within the context of SCI attained the same success. With diagnostic testing, efforts to support local processing of biological samples (e.g., blood/bone marrow immunophenotyping) were met with logistical challenges at some sites, despite best efforts, which necessitated pivoting and incorporating different means to obtain essential diagnostic testing in a timely manner (e.g., international sample analysis).

### Optimized versus routine practices

An integral component of SCI was reviewing routine practices and identifying potential changes to improve patient outcomes and better fit setting-relevant contextual factors. Informed by case presentations and expert opinions, the development of locally-contextualised best practice clinical and supportive care guidance documents was one way this was achieved. The clinical care guidance documents were specific to different types of childhood cancer, outlining treatment recommendations, whereas the supportive care guidance documents focussed on preventing/managing complications due to cancer or treatments. Both guidance types were tailored to the unique resource limitations in the Caribbean context. Similarly, the incidence, treatment, and outcome data collected within the local oncology databases were considered with demographic and disease characteristics, to further understand and inform continuous improvement to clinical and supportive care. Reflection on practices and outcomes, and collecting corresponding data led to implementing meaningful changes.

## Discussion

Successful partnerships between countries with different resource levels can serve as a springboard for systematic improvement to local patient care delivery. Prior to SCI, paediatric haematology/oncology was underserved in the English-speaking Caribbean, but through collaboration there have been significant improvements in the partner countries. Key outcomes of the SCI partnership have included increasing the number of trained local physicians and nurses^21^; improving healthcare professionals' ability to diagnose, treat, and manage complex cases^20^; facilitating world-spanning paediatric haematology/oncology collaborations^23^; developing a system of routine paediatric oncology data collection across participating SCI sites^22^; and cataloguing regional cancer medicine availability, cost, and access barriers.^8^ Heading into the future, both maintaining and further developing SCI will be necessary to ensure sustainability, including continued capacity building through education, adjusting practices to meet local resources and needs, and building research programs. The model of this partnership can be readily applied to other specialities in resource-limited settings, as it highlights the important components such as situational analysis, frank and open dialogue among partners and stakeholders, setting co-developed achievable goals, and building capacity to foster a sustainable health care system.

Several factors facilitated the successes observed within SCI. Importantly, SCI was structured on engaging ethically and equitably to improve health. Priorities were co-developed, and respect was shown towards local culture and values. All partners, local and international, had a shared vision and goal: the advancement of paediatric haematology/oncology care in the partner Caribbean countries through capacity building; accurate and timely diagnoses; dedicated entry and tracking of cases in local databases; and development of critical human resources. Mutual trust and camaraderie underpinned the solid foundation between the Canadian and Caribbean partners. Reflecting on the Canadian Coalition of Global Health Research Principles for Global Health Research,^27^ the SickKids personnel who conceptualised SCI had origins in, or connections with, the Caribbean allowing for easy communication and understanding of how local conditions can impact care delivery. The ability of all stakeholders to articulate their needs and what would feasibly work in the different country-specific settings fostered community (principles: authentic partnering, responsiveness to causes of inequalities). Annual work planning meetings and conducting monitoring and evaluation further supported trust and camaraderie. The objectives of SCI were clearly outlined and reviewed at regular checkpoints to ensure they were met, and communication and discussion were facilitated if issues arose (principles: shared benefits, humility). Importantly, Caribbean partners generated their own objectives for the initiative, which, in turn, were openly discussed with SickKids partners and facilitated in a fair and non-judgemental way at annual meetings via an independent moderator (principle: inclusion). All these factors have collectively been key in fostering local leadership and ownership of SCI and commitment to the future.

Given the unique characteristics of each SCI partner country, developing a single model of care that worked for all six countries was unlikely. Barriers included differences in locally available financial support, population size, access to expertise, laboratory services, pharmaceutical/drug access, and governmental regulations. This led to variations in timelines and feasible accomplishments across the participating countries. For example, while there have been major strides in the care and outcomes for children with cancer and blood disorders in some of the larger SCI countries (e.g., Barbados, The Bahamas, Trinidad and Tobago, Jamaica), the smaller countries have not experienced corresponding magnitudes of improvements (e.g., St. Lucia, St. Vincent and the Grenadines). This can be largely attributed to resource constraints, both on the financial and supply sides. Generally, efforts to strengthen local infrastructure via governments have been slow, although there have been clear, significant gains through SCI.

Interventions related to nursing focussed primarily on strengthening clinical competence through the development and implementation of a post-registration diploma in paediatric haematology/oncology nursing, as well as continuing professional development sessions.^19^ To support education interventions, SCI advocacy and programmatic efforts also addressed an enabling practice environment. Increased collaboration, between SCI physician leads and nursing graduates, was an important component of an enabling environment and supported improvements in interprofessional practice and elevating the role of nursing among successive cohorts of participants. Despite advocacy of the relevant agencies (e.g., hospital boards, Ministries of Health), some challenges affecting paediatric haematology/oncology care within the region could not be addressed within SCI. This included elements related to nursing, scope of practice, and local infrastructure (e.g., provision of hospital-based isolation rooms), which are managed by governmental bodies. Some nurses who received specialised paediatric haematology/oncology training were unable to fully use their acquired skills due to factors in the practice environment, including competing priorities, being reassigned to practice outside the paediatric wards, being promoted, and negotiating tasks with other health care professionals. Targeted quality improvement projects^19^ and development of supportive care guidance documents in collaboration with SCI helped promote evidence-based practice, but there are still opportunities to implement comprehensive policies and procedures for nursing as a strategy to improve the recognition of their specialised skills and scope of practice. Ensuring that nurses can work to their full potential is critical to prevent loss of their specialised skills and migration abroad. One of the key outcomes of SCI was the implementation of interprofessional initiatives, wherein graduate nurses were utilized as coaches/mentors for junior doctors as well as other nurses in clinical settings. This may have contributed to the high retention rates of graduates in the respective countries. There are context-relevant guidelines, which can serve as important tools to support these efforts.^28^

Regarding infrastructure, access to reliable radiological and pathological services and medical supplies was inconsistent. Considering SCD, for example, personnel training at one site to use heel prick and specialised high performance liquid chromatography testing was difficult, given limited local buy-in at the hospital- and/or Ministry of Health-level. In other cases, the paucity of local infrastructure presented a large enough barrier that access to necessary resources (e.g., radiology, pathology) were provided through SickKids. This is not sustainable, and efforts to transform these temporary supports into enduring and locally sustainable clinical programs are a crucial part of ensuring health system capabilities over the long-term. This highlights the ongoing struggles and need for continued advocacy and stakeholder engagement, as resolution of these challenges awaits further effort.

The direct and indirect impact of SCI in the partner countries has been far-reaching. On the direct side, the increase in numbers of certified paediatric haematologists/oncologists has been a major achievement. The data collected within SCI has also led to novel clinical findings. For example, children with acute myeloid leukaemia (AML) were recognised as having poor outcomes across the SCI sites. An AML Working Group was therefore established to facilitate early discussion of newly diagnosed AML patients, between the managing local team and local and international experts. Although formal analyses are ongoing, initial findings suggests a trend towards a decrease in therapy-related mortality rates since these efforts were initiated. In highlighting the predicament of children with cancer and blood disorders in the region, a strong advocacy platform at the local, regional, and international level has also been established, including collaborations with the American Society of Haematology and the World Federation of Haemophilia.

### Future directions

With the completion of phase 2 in March 2022, SCI has shifted towards a regionally led stage of sustainability. This has meant the decentralization of management from Canada to the SCI Caribbean sites, with some shared administrative responsibilities and ongoing case management support. To maintain the vision of SCI, empower local governance, and impact other countries throughout the Caribbean, the emphasis will be on creating a Caribbean paediatric haematology/oncology specialist-led culture of excellence. Achieving this will require dedicated local funding, coupled with continuation and enhancement of the SCI-based successes, such as data analysis, evaluation, strategic planning, engagement with Ministries of Health and regional healthcare agencies, and defined partnerships with international agencies and societies.

To have the greatest impact possible for children <18 years with cancer and blood disorders in the region, leaders in the Caribbean will need to overcome site-specific differences and pursue a unified solution to existing challenges. Three top priorities going forward include (1) engaging Ministries of Health and governmental bodies to improve regional access to resources (e.g., essential drugs, certain diagnostic testing) and care; (2) continuing the paediatric haematology/oncology fellowship program to align the number of specialist physicians with local caseloads; and (3) use the local paediatric oncology data to continuously inform and improve treatment. Given the complexity of some paediatric cancer and blood disorder clinical presentations, ongoing case consultations with SickKids experts will be important. To enhance the gains of SCI, local consideration should also be given to support the training of key allied health professionals, including pharmacists, radiation therapists, and surgical oncologists.

Through consistent efforts and dedication to the improvement of the lives of Caribbean children with cancer and blood disorders, the promise with which SCI started has, in large part, been realised. The initiative will continue to mature, helping these children to realize their full potential.

## Contributors

MRM, CB, and JBB wrote the original draft of the manuscript, with input from VB. All co-authors provided input on the key activities and themes. All other (UA, CA, CBF, JC, SdY, AD, KD, BFC, TG, SG, JKM, MMK, SMS, ONO, KO, SR, CSQ, BS, MT, GW, JW, and SZ) authors reviewed and edited the original draft, and approved the final draft.

## Editor note

The Lancet Group takes a neutral position with respect to territorial claims in published maps and institutional affiliations.

## Declaration of interests

The authors (MRM, CB, JBB, UA, CA, CBF, JC, SdY, AD, KD, BFC, TG, SG, JKM, MMK, SMS, ONO, KO, SR, CSQ, BS, MT, GW, JW, SZ, and VB) declare no competing interests.
